# Impact of COVID-19 on the Surrounding Environment of Nursing Home Residents and Attitudes towards Infection Control and Oral Health Care among Nursing Home Staff in Japan

**DOI:** 10.3390/jcm12051944

**Published:** 2023-03-01

**Authors:** Rena Hidaka, Koichiro Matsuo, Tomoka Maruyama, Kyoka Kawasaki, Itsuki Tasaka, Masami Arai, Satoshi Sakoda, Kazunori Higuchi, Erina Jinno, Tsuyoshi Yamada, Shunsuke Minakuchi

**Affiliations:** 1 Department of Oral Health Sciences for Community Welfare, Graduate School of Medical and Dental Sciences, Tokyo Medical and Dental University, 1-5-45 Yushima, Bunkyo, Tokyo 113-8549, Japan; 2 M’s Dental Clinic, 3F Kitashinagawa 21 Building, 1-1-15 Kitashinagawa, Shinagawa, Tokyo 140-0001, Japan; 3 Sakoda Dental Clinic, 6F Li-Ka 1920, 19-40 Chuoumachi, Kagoshima, Kagoshima 890-0053, Japan; 4 Minnano Dental Clinic, 1F Cainzmall, 3-152 Nijinooka, Tokoname 479-0849, Japan; 5 White Smile Dental Clinic, 2F Mituinoritake Building, 1-4-15 Noritake, Chuo, Nagoya 453-0014, Japan; 6 Shinshiraoka Oral Rehabilitation & Dental Clinic, 7-14-14 Shinshiraoka, Shiraoka 349-0212, Japan; 7 Department of Gerodontology and Oral Rehabilitation, Graduate School of Medical and Dental Sciences, Tokyo Medical and Dental University, Tokyo 113-8549, Japan

**Keywords:** COVID-19, nursing home, infection control, oral health care

## Abstract

The environments of nursing home staff and residents have dramatically changed since the onset of the COVID-19 pandemic, with greater demand for infection control. This study aimed to clarify the changes and regional differences in the surrounding environment of nursing home residents as well as the working environment of staff, including oral health care, after the spread of SARS-CoV-2. A self-administered questionnaire survey was sent to nursing staff at about 40 nursing homes in different areas of Japan in September and October 2021. The questionnaire consisted of items centered around: (1) the surrounding environment of nursing home residents, (2) awareness and attitudes towards daily work among staff, and (3) attitudes to and procedures for oral health care among staff. A total of 929 respondents included 618 (66.5%) nursing care workers and 134 (14.4%) nurses. Regarding changes in resident daily life, 60% of staff perceived decreases in psychosocial and physical function after the start of the pandemic due to limited family communication and recreational activities, especially in urban areas. Concerning infection control, most respondents adopted routines of disinfecting hands before and after their duties. Oral health care was part of the regular duties of over 80% of respondents. Many participants answered that the frequency and time of oral health care only slightly changed after the onset of COVID-19, but many also reported disinfecting hands both before and after oral health care, particularly in rural areas. Our findings suggested that the COVID-19 pandemic decreased the daily living activities of residents, leading to psychosocial and physical decline, especially in urban areas. The results also indicated that the spread of SARS-CoV-2 triggered improvements in the awareness and attitudes towards infection control in daily work, including oral health care, among nursing care staff, notably in rural areas. Such an effect may contribute to a more positive perception of oral health care infection measures after the pandemic.

## 1. Introduction

The spread of the novel coronavirus infection has drastically changed daily life in the medical and nursing care setting since the beginning of 2020 [1,2,3]. In particular, older adults with systemic disorders have a higher mortality risk from COVID-19 [4,5,6,7]. Since COVID-19 was easily transmitted by asymptomatic pathogen carriers, its complete prevention was a struggle in nursing care facilities, which led to mass infection of frail older adults. To reduce the risk of COVID-19 in nursing home residents, various restrictions, including limiting visits of relatives and friends in addition to less frequent recreational activities, have been enforced after the onset of COVID-19 [8].

In addition to residents, nursing home employees have been required to follow thorough control measures to prevent the spread of infection in the event of an outbreak. Oral care procedures are considered high risk for COVID-19 spread due to the direct contact of the operator with the inside of the mouth and the oral secretions that spread outside the mouth during care. Indeed, all related staff have had to be more careful while providing oral health care for residents, with the provision of oral care with adequate infection control measures being recommended by the Ministry of Health, Labour and Welfare of Japan on 30 July 2021 [9]. However, a lack of masks and additional medical equipment hindered the proper implementation of oral care [10]. 

Since the spread of severe acute respiratory syndrome coronavirus 2 (SARS-CoV-2), the circumstances of residents and employees in nursing care facilities have dramatically changed, which may have exacerbated mental stress and increased duties and burden, respectively. Such alterations may also differ between urban, suburban, and rural areas since larger cities suffered higher numbers of infection, and so the atmospheres of care facilities in the face of COVID-19 might have varied. Therefore, we conducted a questionnaire survey of nursing care facility staff in different areas of Japan to clarify the changes in environments and infection control, including oral health care procedures, among residents and staff caused by the spread of SARS-CoV-2.

## 2. Methods

### 2.1. Study Design and Participants

A questionnaire survey was sent to staff working at 40 long-term nursing homes in Tokyo, Aichi and Kagoshima prefectures as representatives of urban, suburban, and rural areas of Japan, respectively, in September and October 2021 following study protocol approval by the ethics committee of Tokyo Medical and Dental University (D2021-038). The survey was administered by mail as a self-administered, unsigned questionnaire, an explanatory document describing the purpose of the study, and a consent form. Respondents were regarded as agreeing with participation in the study by submitting their answers. The survey was administered both on paper and online, giving respondents options on the mode of questionnaire completion. Completed questionnaires were returned using an enclosed return envelope or via the Internet.

### 2.2. Questionnaire

**Figure 1:**
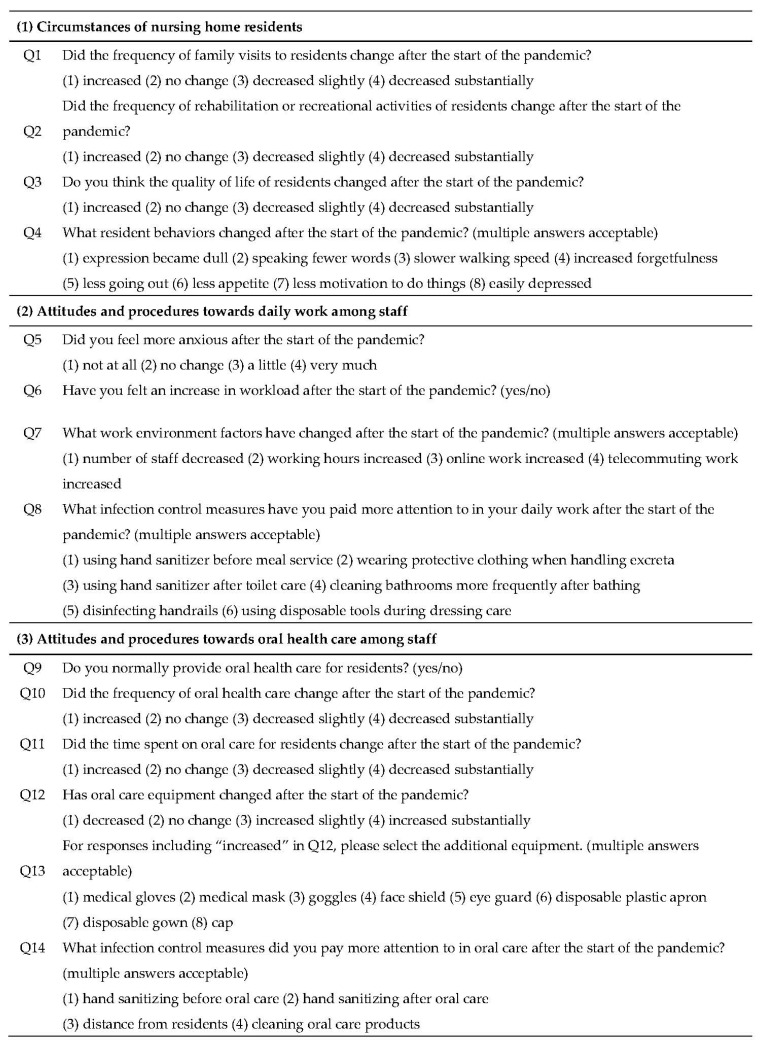
Questionnaire (translated from Japanese).

We asked staff about the environment at nursing homes following the spread of SARS-CoV-2 up to October 2021. The questionnaire was developed based on the Guideline for Infection Control in Nursing Care Settings from the Ministry of Health, Labour and Welfare of Japan (second edition, revised 30 July 2021) [9,11] (Figure 1). The survey included items related to changes in (1) the surrounding environment of nursing home residents, (2) awareness and attitudes towards daily work among staff, and (3) attitudes and procedures of oral health care among staff after the spread of SARS-CoV-2. More specifically, the questionnaire aimed to clarify the following items:

(1) Surrounding environments of nursing home residents: items included the frequency of family visits, staff communication, and rehabilitation and recreation, as well as quality of life and activities of daily living. 

(2) Awareness and attitudes towards daily work among staff: items included changes in workload before and after the spread of infection, work precautions, and concerns. 

(3) Attitudes and procedures of oral health care: items included changes in infection control, equipment, and time and frequency of performing oral care.

### 2.3. Statistical Analysis

Each survey item was summarized by region using descriptive statistics. Regional differences were examined by means of the χ^2^ test, and significance levels were adjusted using the Bonferroni method. All statistical analyses were performed using SPSS 25.0 software (IBM Corp., Armonk, NY, USA). A *p*-value of <0.05 was considered statistically significant. 

## 3. Results

### 3.1. Participant Characteristics

Completed questionnaires were returned from 929 nursing care staff (313 men [33.7%]) of 40 nursing homes. By region, 200 respondents (21.5%) were from urban areas, 547 (58.9%) were from suburban areas, and 182 (19.6%) were from rural areas. By age group, respondents in their forties accounted for the largest number (280, 30.4%), followed next by those in their thirties (205, 22.1%) and in their fifties (195, 21.0%). The most common respondent job position was ‘nursing care worker’ (618, 66.5%), followed next by ‘nurse’ (134, 14.4%) and ‘care manager’ (38, 4.1%). 

### 3.2. Surrounding Environment of Nursing Home Residents 

More than 70% of respondents reported that the frequency of family involvement with facility residents had decreased, which tended to be more prevalent in urban areas than in suburban areas (Q1; *p* = 0.052 for urban vs. suburban in multiple comparison testing) (Table 1). Approximately 60% of respondents answered that the frequency of recreation and rehabilitation activities decreased after the spread of SARS-CoV-2, which was significantly higher in urban and rural areas than in suburban areas (Q2; *p* < 0.01 in multiple comparison testing). A similar trend was observed for changes in the quality of life of residents in that 69.5% of staff in urban, 52.7% in suburban, and 61.5% in rural areas reported diminished quality of life among residents. This finding was significantly higher in urban and rural areas than in suburban areas (Q3; *p* < 0.05 in multiple comparison testing). 

Table 2 shows the changes in resident behavior after the spread of the pandemic (Q4). More than 60% of respondents reported that facility residents no longer went out. Approximately 25% responded that residents showed a lack of facial expression, increased time in bed, and increased forgetfulness, although the response rates exhibited regional differences.

### 3.3. Awareness and Attitudes towards Daily Work among Staff 

For Q5, about anxiety in their lives, more than 60% of respondents reported that their sense of anxiety ‘increased’ after the spread of SARS-CoV-2, with no significant regional differences (*p* = 0.219). We observed that 62.5% of respondents described an overall increase in workload after the onset of COVID-19 (Q6). Twenty-nine percent of staff reported more online work, the percentage of which was particularly high in rural areas (Q7) (Table 3). Approximately 15% responded that the number of staff decreased, particularly in urban areas (26.9%) over other areas (*p* < 0.001 for urban vs. suburban and *p* < 0.001 for urban vs. rural). Telecommuting work did not increase remarkably in any region.

Items related to infection control, such as sanitizing hands and wearing masks, were cited as receiving increased awareness in daily work (Q8) (Table 4). Attention to infection control increased significantly more in rural areas than other areas after the start of the pandemic.

### 3.4. Attitudes and Procedures of Oral Health Care

A total of 744 respondents (80.1%) answered to be involved in oral health care work in Q9. We witnessed that 95.9% and 95.2% reported ‘no change’ in the frequency and time spent on oral health care procedures (Q10 and Q11, respectively). More than 70% of participants cited ‘no change’ in the equipment used for oral health care after the infection spread in Q12. Approximately 10% described the addition of equipment, including face shields, medical gloves, and medical masks (Q13). Regarding infection control for oral health care procedures, hand sanitizing ‘before’ and ‘after’ oral health care tended to be selected most frequently by roughly 60% of respondents each, especially in rural areas (roughly 80%). This was followed next by ‘cleaning oral care products (42.3%)’ and ‘distance from the patient (23.9%)’ (Q14) (Table 5). 

## 4. Discussion

In this study, answers from staff members suggested several significant changes in the surrounding environment of nursing home residents as well as in the working environment, including oral health care, of nursing home staff after the spread of SARS-CoV-2. Many of these alterations differed among urban, sub-urban, and rural settings. Our findings showed that many staff recognized a negative impact of COVID-19 on the activities of daily life of facility residents. However, there was also a possible positive influence on the awareness and attitudes towards infection control in oral health care and other daily work among staff, as significant increases in attention to infection control measures in daily work and oral health care were observed. 

### 4.1. Differences in the Changes in Attitudes of Nurisng Care Staff in Different Areas of Japan

We observed significant differences in survey answers among urban, suburban, and rural areas. Our findings suggested that nursing care staff perceived that the residents’ living environments deteriorated significantly, and more so in urban centers than in other locations. However, attention to infection control at work, including oral health care, changed significantly more in rural areas than elsewhere. Since April 2020, a state of emergency has been declared multiple times in Japan, but many restrictions depended on the area and circumstances of COVID-19 spread. According to a survey by the Cabinet Office of Japan [12], there has been an increased desire to move to rural areas among urbanites after the spread of infection and a change in commuting time with the introduction of remote work. Urban residents have reported greater anxiety and poorer mental health related to COVID-19, which may have influenced regional differences [13,14,15].

### 4.2. Surrounding Environments of Nursing Home Residents

Many staff cited that the surrounding environments of nursing home residents became restricted after the spread of SARS-CoV-2. The decreased access to residents’ family visits and recreation activities may have impacted them both psychosocially and physically. A longer bedridden status may have increased the risk of frailty and sarcopenia [16,17]. In addition to physical frailty, the prohibition of family visits and reduced group recreation at institutions could have reduced psychosocial functioning [18,19,20]. Our findings suggest that the reduced activity levels caused by the COVID-19 pandemic had a strong impact on the activities of daily living and quality of life of institution residents. 

### 4.3. Awarenesss and Attitudes towards Daily Work among Staff

Many respondents reported an overall increase in workload. Nursing care facilities are chronically understaffed [21]. In addition to a rise in COVID-19-related work, absenteeism due to coronavirus infection may have caused work to be transferred to the remaining staff members [22]. A recent study revealed that nursing home staff were more likely to leave their jobs under the complex and stressful circumstances of prolonged SARS-CoV-2 infections [23]. Indeed, the pandemic significantly influenced the working situation in nursing care facilities. 

Respondents replied that online communication increased significantly, especially in rural areas, which indicated that the nature of their work had changed. Since restricting interactions with the outside world was described as effective in preventing SARS-CoV-2 transmission [24], many nursing care facilities shifted to online interaction tools for residents to see their families instead of face-to-face contact after the start of the pandemic. Our findings suggested that many facilities in rural areas adopted online tools after the pandemic onset. 

Institutional attitudes towards infection control have dramatically changed since the start of COVID-19 [25]. Our findings showed that a high percentage of respondents paid more attention to infection control in their daily work. Since the transmission of COVID-19 is often asymptomatic, the difficulty of infection control measures raised anxiety levels among staff [26]. The rapid spread of SARS-CoV-2 infection may have augmented the awareness and attitudes towards disinfection in all areas of Japan as compared with beforehand. 

### 4.4. Attitudes and Procedures of Oral Health Care

Our findings indicated that the awareness and attitudes towards infection control in oral health care procedures significantly increased after the onset of the pandemic. Approximately 60% of respondents reported sanitizing their hands before and after oral health care, with the higher percentage of these being significantly higher in rural areas. As with other domains of infection control, attitudes to oral health care were also heightened in all areas of Japan. The number of deaths from pneumonia significantly decreased during the pandemic [27]. The commitment to thorough infection control in older adults as well as among nursing care and hospital staff may have contributed to reducing mortality by respiratory infection diseases [28,29,30]. 

Our survey indicated that the procedures of oral health care themselves were not remarkably changed after the spread of SARS-CoV-2. However, staff awareness towards oral health care equipment has changed during the pandemic. Goggles and face shields have been reported to be preferentially worn over eyeglasses since oral health care can increase the risk of droplets entering into the eyes [31,32]. The increased frequency of protective equipment use might have been driven by improvements in knowledge and attitudes towards infection control. 

This study had several limitations. First, the findings were based on the responses of staff members belonging to each facility rather than on a facility-by-facility basis. The answers may therefore have been influenced by the subjectivity of individual respondents. Also, we did not identify the total number of employees of each facility; thus the result may be limited to the opinions of certain employees. Second, due to the need for quickly assessing the influence of COVID-19 in nursing homes, the reliability and validity of the questionnaire may not have been sufficiently evaluated. Third, the risk of confounding factors, selection bias, and recall bias needs to be considered when interpreting the results. Lastly, due to the large number of categorical variables, the possibility of type II error cannot be ruled out. However, this study was conducted swiftly during a pandemic and was therefore considered important in capturing the real-life impact of COVID-19. Future studies should examine the reliability and validity of the questionnaire, increase the number of facilities surveyed, and examine the long-term effects of the changing situation.

## 5. Conclusions

This questionnaire survey of staff in nursing homes across Japan revealed a decline in physical and psychological function of the residents, and an increased awareness of infection control among facility staff members, especially in rural areas. Efforts to maintain good oral hygiene and persistent attention to other infection control measures are being implemented on an ongoing basis.

## Figures and Tables

**Table 1:** Proportion of answers described as “decreased slightly” or “decreased substantially” in frequency of family visits, rehabilitation and recreation, and resident QOL after the start of the pandemic (N, %) (Q1–Q3).

	Total	Urban	Suburban	Rural	*p*-Value
Frequency of family visits	702 (87.2)	167 (83.5)	392 (71.7)	143 (78.6)	0.027 ^b^
Frequency of rehabilitation and recreation	575 (67.9)	144 (76.1)	311 (56.9)	120 (65.9)	0.002 ^ac^
Resident QOL	539 (63.6)	139 (73.5)	288 (58.3)	112 (67.9)	0.002 ^ac^

a, urban vs. suburban; b, urban vs. rural; c, suburban vs. rural. QOL, quality of life.

**Table 2:** Changes in resident behavior after the start of the pandemic (N, %) (Q4).

	Total (881)	Urban (191)	Suburban (517)	Rural (172)	*p*-Value
Physical factors					
Less going out	562 (63.8)	130 (68.0)	319 (61.7)	113 (65.7)	0.252
Slower walking speed	67 (7.6)	24 (12.6)	33 (6.3)	10 (5.8)	0.014 ^ab^
Psychological factors					
Expression became dull	243 (27.5)	67 (35.0)	116 (22.4)	60 (34.9)	<0.001 ^a^
Less motivation to do things	217 (24.6)	55 (28.8)	118 (22.8)	44 (25.6)	0.244
Speaking fewer words	221 (25.1)	49 (25.6)	124 (24.0)	48 (27.9)	0.570
Increased forgetfulness	180 (20.4)	58 (30.4)	96 (18.6)	26 (15.1)	<0.001 ^ab^
Easily depressed	89 (10.1)	29 (15.2)	46 (8.9)	14 (8.1)	0.030 ^ab^
Nutritional factors					
Less appetite	96 (10.8)	23 (12.0)	44 (8.5)	29 (16.8)	0.008 ^c^

a, urban vs. suburban; b, urban vs. rural; c, suburban vs. rural.

**Table 3:** Changes in work environment after the start of the pandemic (N, %) (Q7).

	Total (871)	Urban (156)	Suburban (535)	Rural (180)	*p*-Value
Online work increased	250 (28.7)	37 (23.7)	146 (27.3)	67 (37.2)	0.010 ^c^
Number of staff decreased	120 (14.8)	42 (26.9)	68 (12.7)	19 (10.5)	<0.001 ^ab^
Working hours increased	111 (12.7)	29 (18.6)	57 (10.7)	25 (13.9)	0.029 ^a^
Telecommuting work increased	1 (0.1)	0 (0)	1 (0.2)	0 (0)	0.730

a, urban vs. suburban; b, urban vs. rural; c, suburban vs. rural.

**Table 4:** Increased attention to infection control measures after the start of the pandemic (N, %) (Q8).

	Total (925)	Urban (196)	Suburban (547)	Rural (182)	*p*-Value
Using hand sanitizer after toilet care	657 (71.0)	129 (65.8)	383 (70.0)	145 (79.6)	0.010 ^bc^
Disinfecting handrails	607 (65.6)	129 (65.8)	348 (63.6)	130 (71.4)	0.156
Using hand sanitizer before meal service	579 (62.6)	118 (60.2)	327 (59.8)	134 (73.6)	0.003 ^bc^
Wearing protective clothing when handling excreta	149 (16.1)	42 (21.4)	56 (10.2)	51 (28.0)	<0.001 ^ac^
Frequent cleaning of bathrooms after bathing	138 (14.9)	50 (25.5)	53 (9.7)	35 (19.2)	<0.001 ^ac^
Using disposable tools during dressing care	80 (8.6)	16 (8.1)	36 (6.6)	28 (15.4)	0.001 ^bc^

a, urban vs. suburban; b, urban vs. rural; c, suburban vs. rural.

**Table 5:** Increased infection control measures in oral health care after the start of the pandemic (N, %) (Q14).

	Total (829)	Urban (163)	Suburban (500)	Rural (166)	*p*-Value
Hand sanitizing after oral care	519 (62.6)	106 (65.0)	278 (55.6)	135 (81.7)	<0.001 ^bc^
Hand sanitizing before oral care	470 (56.7)	53 (32.5)	248 (49.6)	129 (77.7)	<0.001 ^bc^
Cleaning oral care products	351 (42.3)	75 (46.0)	194 (38.8)	82 (49.3)	0.039 ^bc^
Distance from the patient	198 (23.9)	52 (31.9)	102 (20.4)	44 (26.5)	0.007 ^a^

a, urban vs. suburban; b, urban vs. rural; c, suburban vs. rural.

## Data Availability

The data presented in this study are available on request from the corresponding author. The data are not publicly available due to the protection of personal data.
